# Supplementary figures and images for: The Immunomodulatory Effect of Different FLT3 Inhibitors on Dendritic Cells
Source: Cancers (Basel). 2024 Nov 4;16(21):3719. doi: 10.3390/cancers16213719 (PMC11545830; doi:10.3390/cancers16213719)

# Gel1: c-Rel (78 kDa)

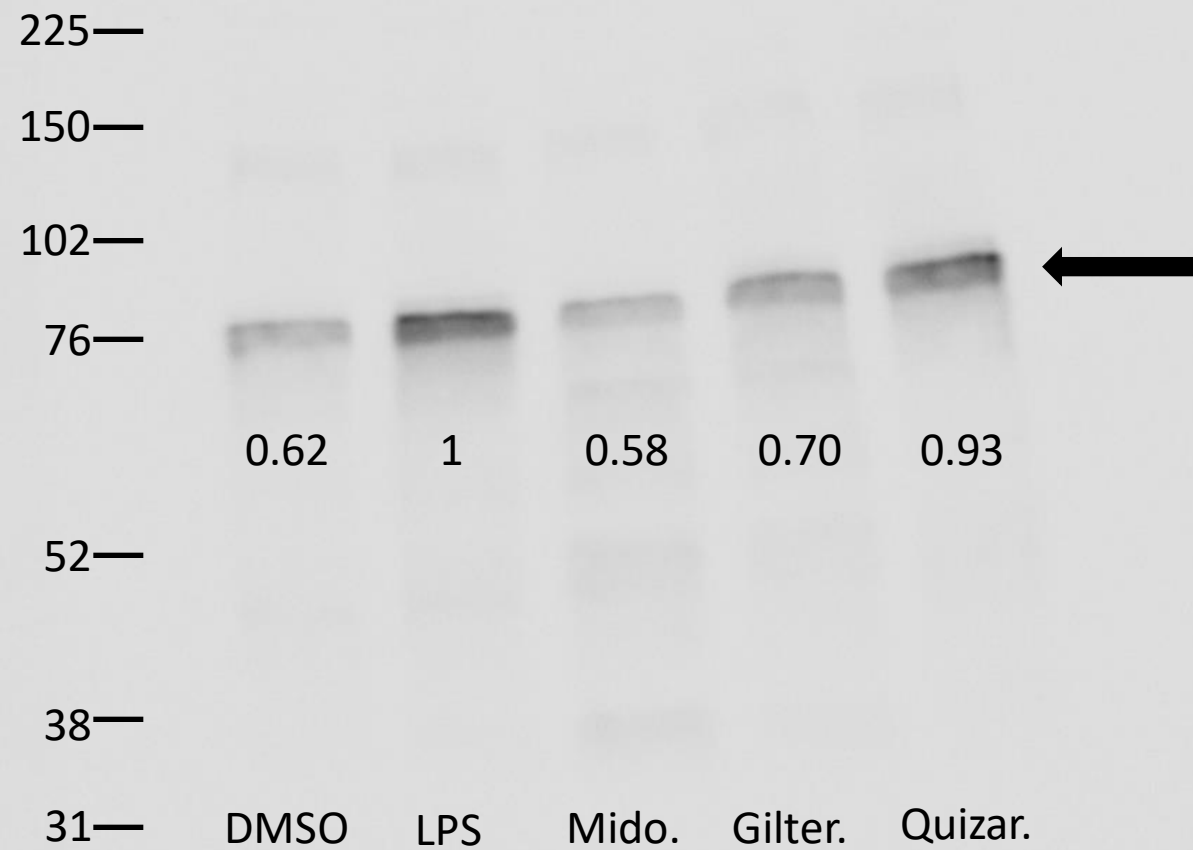

# Gel1: RelB (70 kDa)

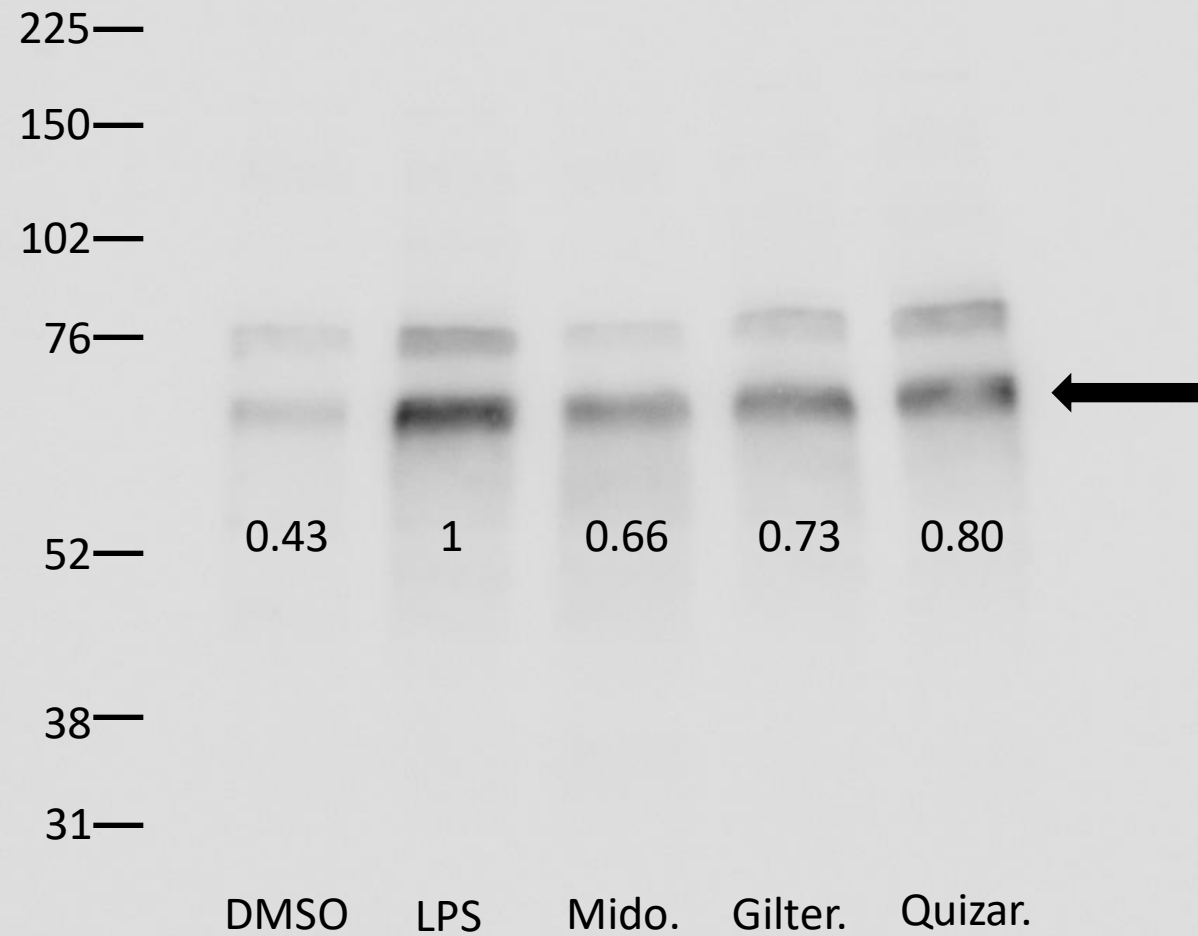

# Gel1: GAPDH (37 kDa)

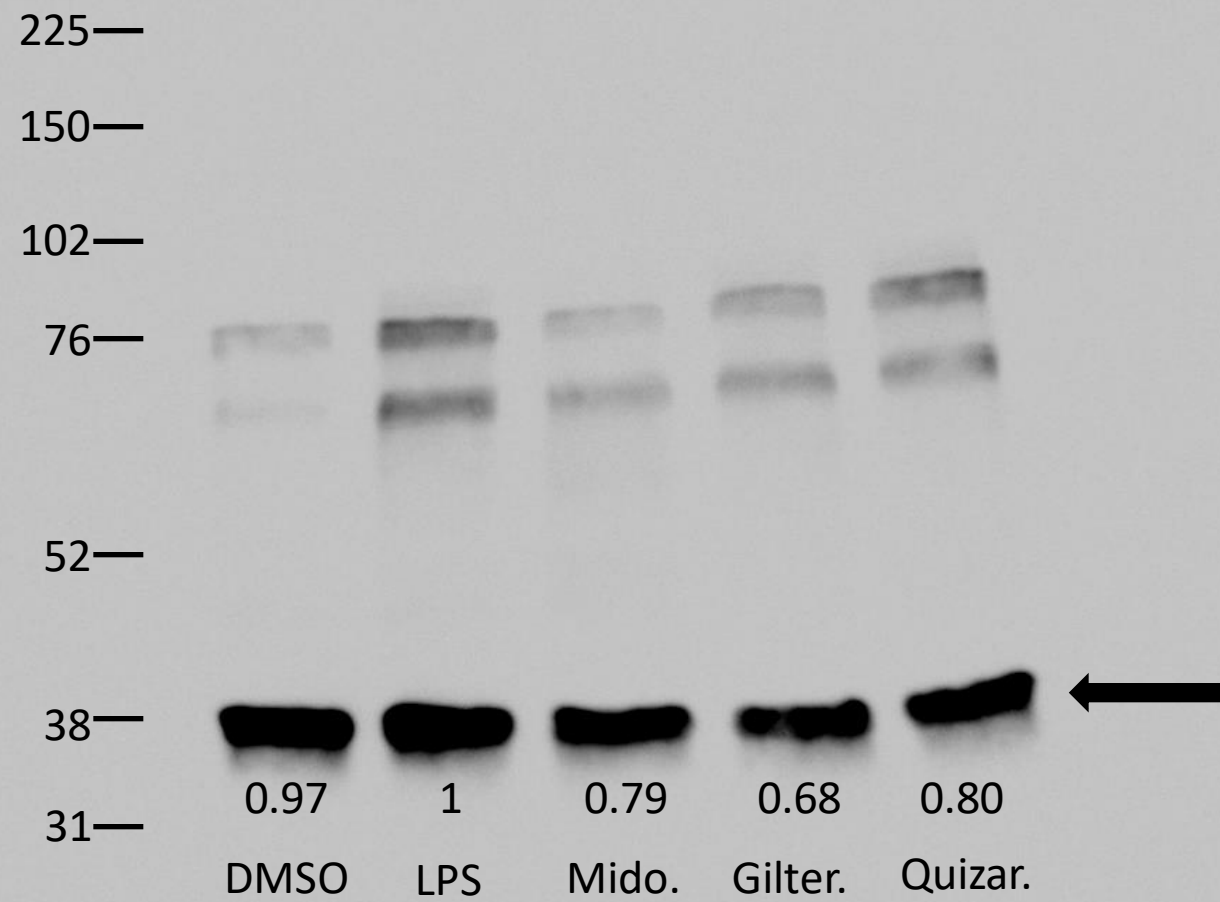

Supplement: Supplementary file 1 [file cancers-16-03719-s001.zip › Gel1.pdf]

# Gel2: pSTAT5 (90 kDa)

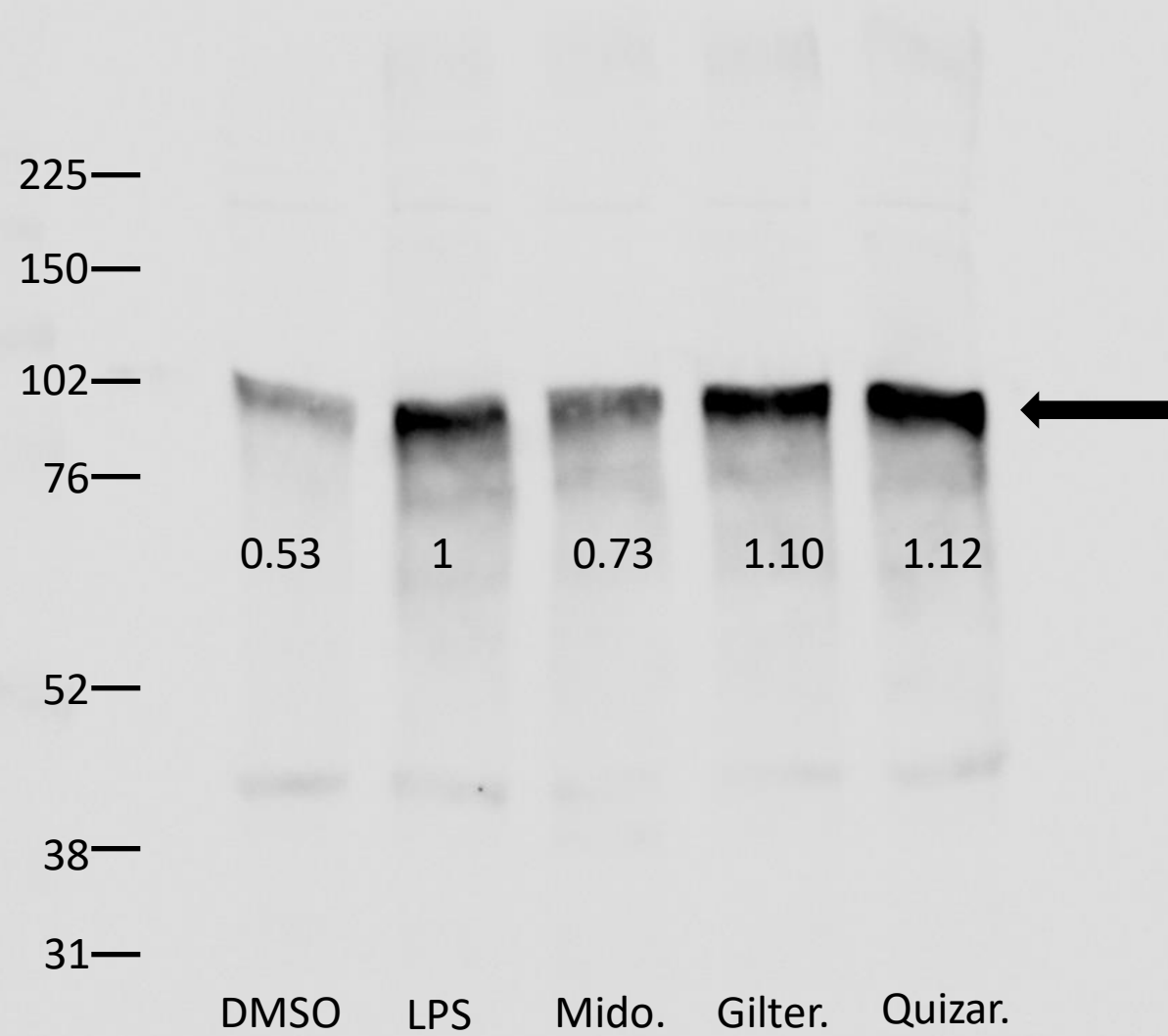

# Gel2: pSTAT3 (79, 86 kDa)

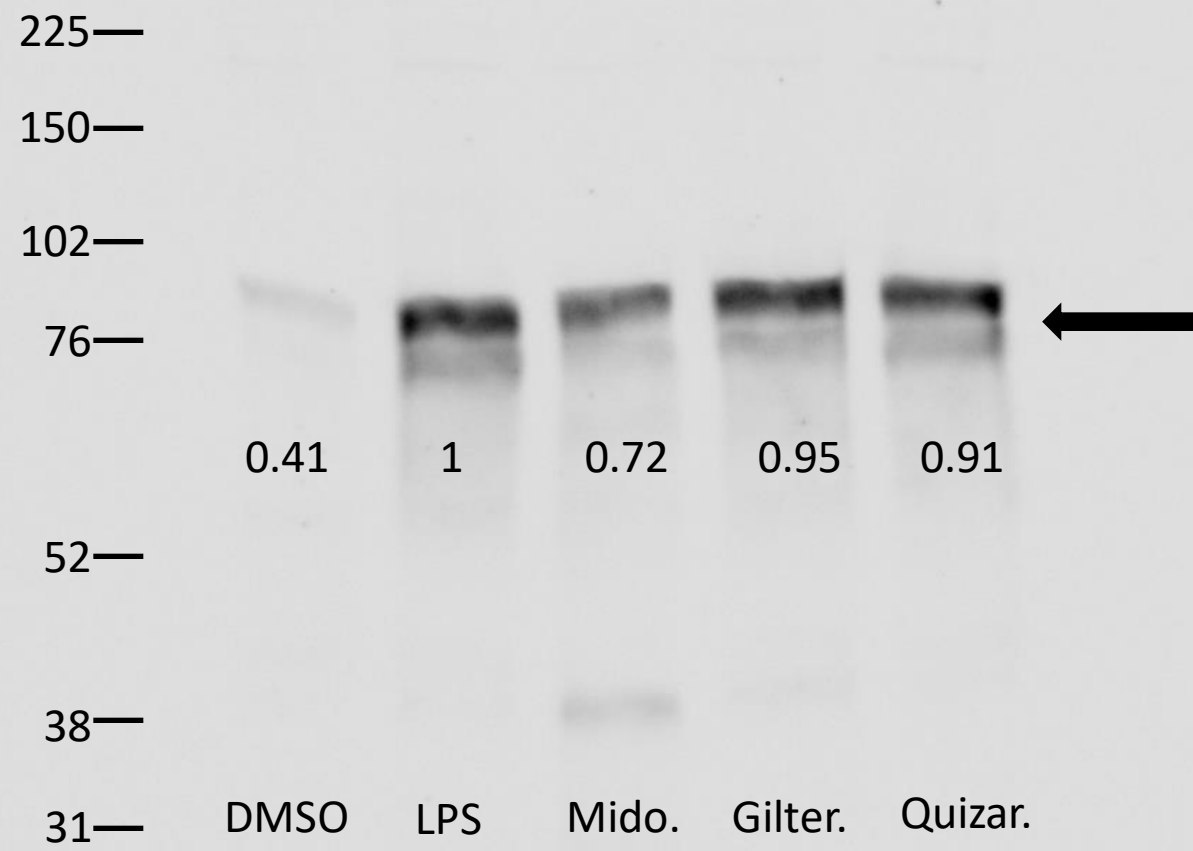

# Gel2: GAPDH (37 kDa)

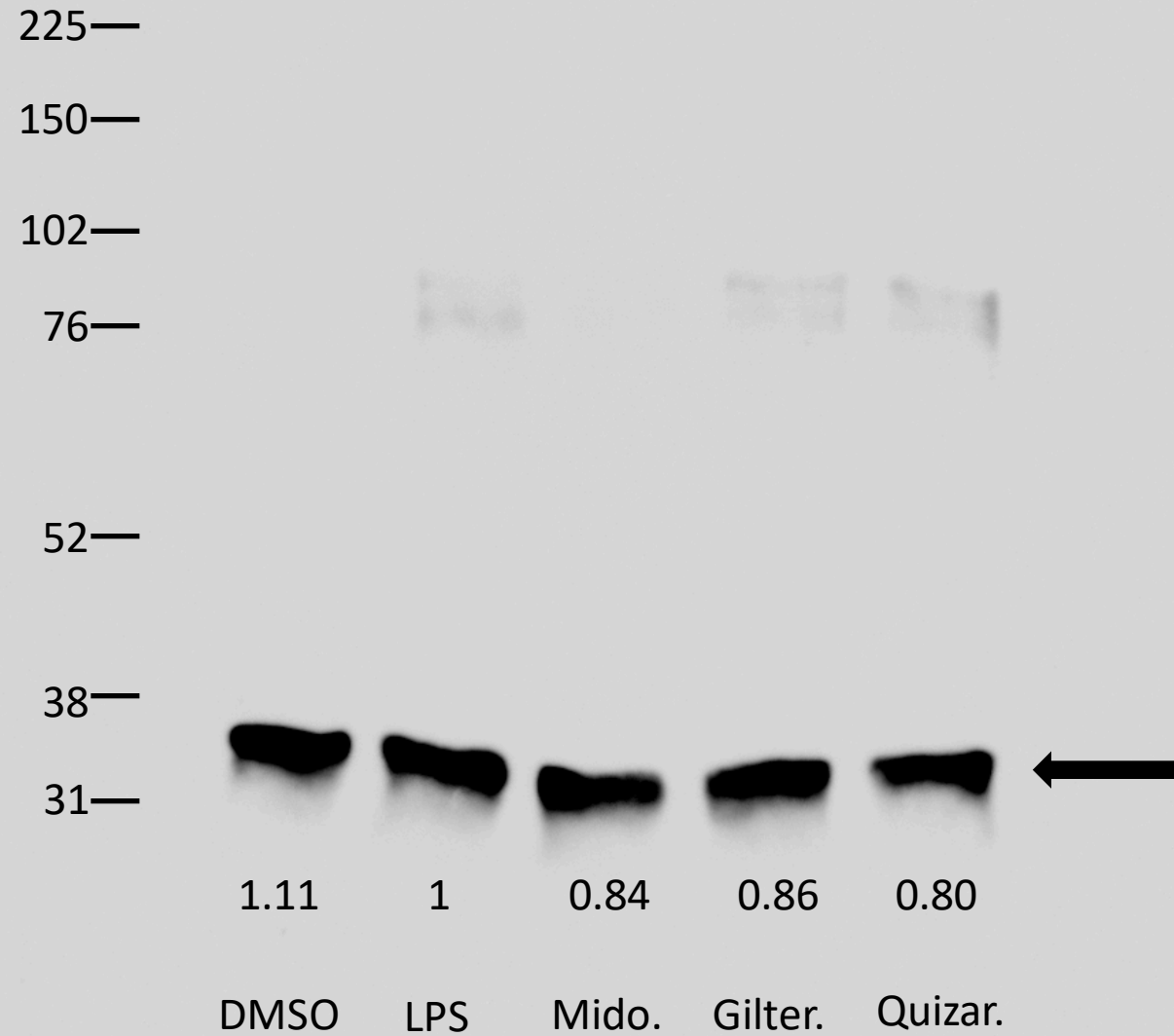

Supplement: Supplementary file 1 [file cancers-16-03719-s001.zip › Gel2.pdf]

# Gel3: pAkt (60 kDa)

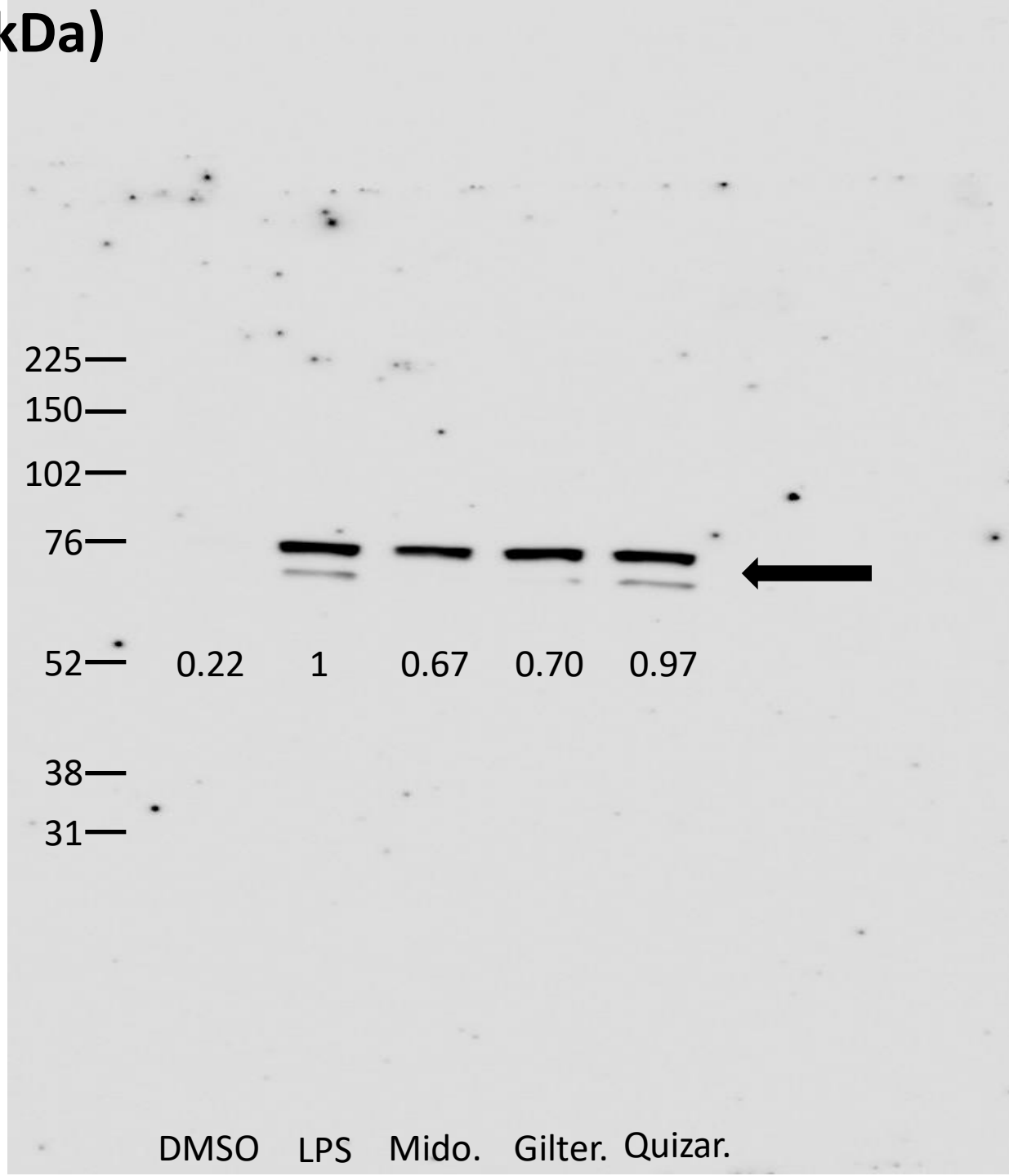

# Gel3: GAPDH (37 kDa)

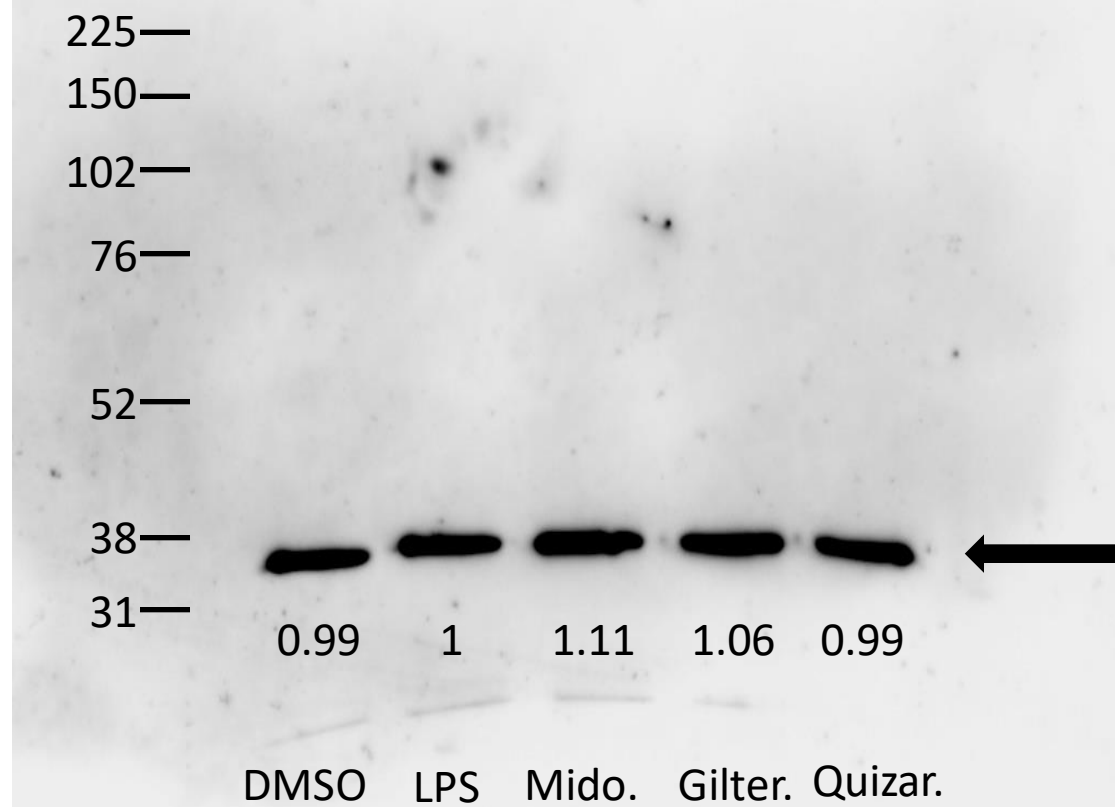

Supplement: Supplementary file 1 [file cancers-16-03719-s001.zip › Gel3.pdf]
